# *Drosophila* protease ClpXP specifically degrades DmLRPPRC1 controlling mitochondrial mRNA and translation

**DOI:** 10.1038/s41598-017-08088-6

**Published:** 2017-08-16

**Authors:** Yuichi Matsushima, Yuta Hirofuji, Masamune Aihara, Song Yue, Takeshi Uchiumi, Laurie S. Kaguni, Dongchon Kang

**Affiliations:** 1 Department of Clinical Chemistry and Laboratory Medicine, Graduate School of Medical Sciences, Kyushu University, 3-1-1 Maidashi, Higashi-ku, Fukuoka 812-8582 Japan; 2 Department of Biochemistry and Molecular Biology, and Center for Mitochondrial Science and Medicine, Michigan State University, East Lansing Michigan, 48824-1319 USA; 3 Section of Pediatric Dentistry, Division of Oral Health, Growth and Development, Faculty of Dental Science, Kyushu University, 3-1-1 Maidashi, Higashi-ku, Fukuoka 812-8582 Japan

## Abstract

ClpXP is the major protease in the mitochondrial matrix in eukaryotes, and is well conserved among species. ClpXP is composed of a proteolytic subunit, ClpP, and a chaperone-like subunit, ClpX. Although it has been proposed that ClpXP is required for the mitochondrial unfolded protein response, additional roles for ClpXP in mitochondrial biogenesis are unclear. Here, we found that *Drosophila* leucine-rich pentatricopeptide repeat domain-containing protein 1 (DmLRPPRC1) is a specific substrate of ClpXP. Depletion or introduction of catalytically inactive mutation of ClpP increases DmLRPPRC1 and causes non-uniform increases of mitochondrial mRNAs, accumulation of some unprocessed mitochondrial transcripts, and modest repression of mitochondrial translation in *Drosophila* Schneider S2 cells. Moreover, DmLRPPRC1 over-expression induces the phenotypes similar to those observed when ClpP is depleted. Taken together, ClpXP regulates mitochondrial gene expression by changing the protein level of DmLRPPRC1 in *Drosophila* Schneider S2 cells.

## Introduction

ClpXP, Lon and *m*-AAA are ATP-dependent proteases that contribute to the degradation of all mitochondrial matrix proteins^1–5^. ClpXP and Lon localize to the matrix space whereas the *m-*AAA protease is anchored in the membrane with its catalytic site exposed to the matrix space. ClpXP is composed of a proteolytic subunit, ClpP, and a chaperone-like subunit, ClpX, which carries an ATPase associated with diverse cellular activities (AAA+) domain^6, 7^.

ClpXP is a barrel-shaped, hetero-oligomeric complex in which ClpP forms a two-stack heptameric ring-shaped structure to which two hexameric ClpX rings bind on each side. Lon also carries an AAA+ domain and forms homo-oligomeric, ring-shaped complexes^8, 9^. Lon and ClpP contain serine protease domains and these proteases are well conserved among species. Interestingly, mutations in each protease cause different diseases. Mutations in the gene encoding ClpP cause sensorineural hearing loss and ovarian failure in Perrault syndrome, while mutations in the genes encoding mitochondrial Lon cause cerebral, ocular, dental, auricular, skeletal anomalies (CODAS) syndrome^10, 11^. Moreover, a ClpP knock out in the mouse results in a phenotype similar to the relatively modest phenotype observed in patients with Perrault syndrome whereas deletion of the mitochondrial Lon protease gene causes early lethality^12, 13^.

ClpXP and Lon function mainly to degrade misfolded or damaged proteins (for example, proteins damaged by oxidation) to prevent the cell from accumulating defective proteins. These protein degradation pathways are collectively called protein quality control^4, 14, 15^. Although there is some overlap in the substrate specificities of mitochondrial proteases, it has been proposed that some mitochondrial matrix proteins are specific substrates for each of them. However, to date, a limited number of proteins has been shown to be specific substrates for each^5, 16^. In addition, ClpXP and Lon also play a key role in mitochondrial biological processes. For instance, we have shown previously that Lon regulates mitochondrial DNA (mtDNA) transcription by regulating the ratio of mitochondrial transcription factor A (TFAM) to mtDNA via degradation of TFAM^5, 17^. In addition, ClpXP modulates mitochondrial unfolded protein responses in *C. elegans* and mammals^18–20^.

Metazoan mtDNA is transcribed as precursor polycistronic RNAs containing rRNAs, tRNAs, and mRNAs^21, 22^. After RNA processing and maturation, each RNA contributes to mitochondrial translation. The pentatricopeptide repeat (PPR) domain is a RNA binding domain and, in metazoans, the PPR protein family participates in mitochondrial RNA biogenesis^21–24^. Leucine-rich pentatricopeptide repeat domain–containing proteins, called LRPPRCs, have multiple PPR domains^25^. Both mammalian LRPPRC and its *Drosophila melanogaster* homologue, DmLRPPRC1 are required for polyadenylation of mitochondrial mRNAs (mt-mRNAs)^26–28^. LRPPRCs also control mt-mRNA stability, as loss of LRPPRC or DmLRPPRC1 results in a non-uniform reduction of mitochondrial mRNA abundance^26–28^. LRPPRC interacts with the non-PPR RNA binding protein, SRA stem-loop interacting RNA binding protein (SLIRP) in ribonucleoprotein complexes^27, 29–31^. Interestingly, LRPPRC and SLIRP are degraded in mitochondria under some experimental conditions such as inhibition of mitochondrial transcription^27, 30, 31^.

In this study, we investigated the role of ClpXP protease in regulating the abundance of mitochondrial mRNA, and processing and translation of mitochondrial RNA in *Drosophila* cells. Our results argue strongly that ClpXP modulates mitochondrial RNA biogenesis by the selective degradation of DmLRPPRC1.

## Resutls

### RNA interference (RNAi)-dependent knockdown of ClpP increases mitochondrial transcription

**Figure 1:**
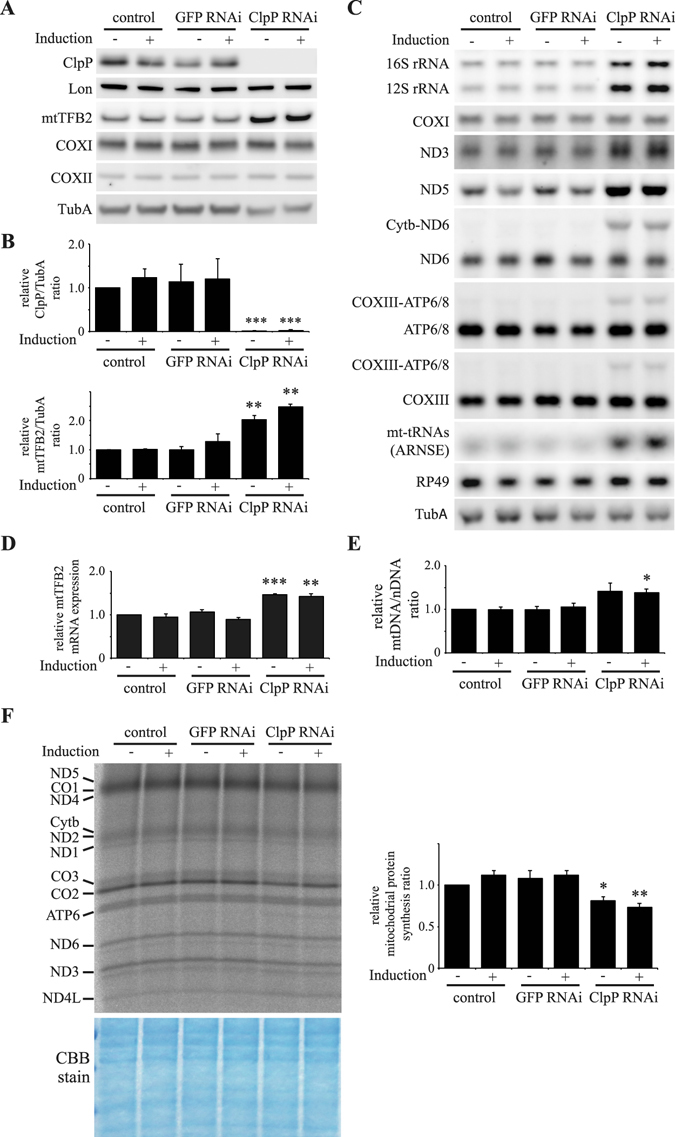
*Depletion* of *Drosophila* ClpP protease in Schneider S2 cells. Schneider S2 cells with no plasmid (control) or carrying pMt/invGFP/Hy (GFP RNAi) or pMt/invClpP/Hy (ClpP RNAi) were cultured for 5 d in the presence or absence of 0.1 mM CuSO4. (**A**) Immunoblot analysis was carried out as described in Materials and Methods. (**B**) Relative protein ratios of ClpP and mtTFB2 was quantitated as described in Materials and Methods. The results represent the mean ± SD of three independent experiments. **P < 0.01; ***P < 0.001 versus control cells without induction (Student’s t-test). (**C**) Northern blot analysis of the levels of mitochondrial genome encoded transcripts in ClpP depleted Schneider S2 cells. The nuclear genome encoded transcripts, *RP49 and TubA*, was used as a loading control. The results are representative of two independent experiments. (**D**) The relative ratio of mtTFB2 mRNA was quantitated as described in Materials and Methods. The results represent the mean ± SD of three independent experiments and **P < 0.01; ***P < 0.001 versus control cells without induction. (**E**) The relative mtDNA copy number was quantitated as described in Materials and Methods. The results represent the mean ± SD of three independent experiments and *P < 0.05 versus control cells without induction (Student’s t-test). (**F**) Mitochondrial translation products were labeled in Schneider S2 cells after 5 d of culture in the presence or absence of 0.1 mM CuSO_4_. Labeling was carried out with EXPRE^35^S^35^S protein labeling MIX for 45 min in the presence of emetine and cycloheximide, and then total cell lysates were prepared. Coomassie Brilliant Blue (CBB) staining was used to check for equal loading of the samples. *Left panel*, aliquots containing 10 μg of total protein each were fractionated by 15–20% gradient SDS-PAGE. *Right panel*, the relative mitochondrial protein synthesis ratio of ClpP-depleted cells. Schneider S2 cell lines were cultured for 5 d in the presence or absence of 0.1 mM CuSO4. The relative mitochondrial protein synthesis ratio was quantitated by average of the radiolabeled products of mtDNA encoded subunits, COX1 and COX2. The results represent the mean ± SD of three independent experiments and *P < 0.05; **P < 0.01 versus control cells without induction (Student’s t-test). Full blots are presented in Supplementary Fig. S9.

To characterize the functional role of *Drosophila* ClpP (CG5045), we adopted a strategy of gene silencing by RNAi in Schneider S2 cells that we reported previously^17, 32–34^. The abundance of ClpP was reduced by expressing a metallothionein-inducible RNA targeting *d-ClpP* from the plasmid pMt/invClpP/Hy. The RNA produced a dsRNA hairpin homologous to *ClpP*. Schneider S2 cells carrying pMt/invClpP/Hy were cultured for 5 d in the absence or presence of 0.1 mM CuSO_4_. Immunoblot analysis of copper-treated cells showed an undetectable level of ClpP in cells carrying pMt/invClpP/Hy (Fig. 1A,B). Even without the addition of copper, ClpP protein is undetectable, most likely due to leaky expression of dsRNA as described previously^17, 32–34^. In contrast, expression of α-tubulin and another mitochondrial matrix protease, Lon, were not changed under any experimental conditions.

To assess the effect of ClpP knockdown in cells, mitochondrial transcript levels and mtDNA copy number were measured. Northern blots were used to quantitate relative expression of mtDNA-encoded genes in cells grown for 5 d in the presence or absence of copper. Depletion of *d-ClpP* resulted in an increase of the transcript levels of 12S rRNA and 16S rRNA, and mtDNA encoded tRNAs relative to their levels in control cells (Fig. 1C). In contrast, the level of transcripts from the nuclear gene, ribosomal protein 49 (*RP49*), was unchanged. Because the steady-state levels of mitochondrial tRNAs are good indicators of *de novo* transcriptional activity^26, 35^, the increase of mitochondrial transcripts in ClpP RNAi cells may result from an increase in mitochondrial transcription. To confirm this, we examined the protein levels of the mitochondrial transcription factor B2, mtTFB2^33^, also known as TFB2M, in ClpP knockdown cells by western blot. In ClpP RNAi cells, protein levels of mtTFB2 were increased more than two-fold relative to control cells (Fig. 1A,B). To assess whether the increase of mtTFB2 is due to an increase in accumulation of the mRNA, the levels of *mtTFB2* mRNA were quantified with a probe-based reverse transcription quantitative PCR assay (RT-qPCR). The analysis showed that the amount of *mtTFB2* mRNA was also increased relative to control cells (Fig. 1D). Taken together, the increase of mtTFB2 protein seems to be the result of an increase in accumulation of the *mtTFB2* mRNA rather than a deficiency in mtTFB2 proteolysis by ClpP depletion. Furthermore, relative mtDNA copy number was increased approximately 1.4-fold in the knockdown cells (Fig. 1E).

### ClpP depletion causes non-uniform increases of mitochondrial mRNA abundance and unprocessed mRNAs

Interestingly, northern blot analysis demonstrated that the levels of mitochondrial mRNAs (mt-mRNAs) in ClpP RNAi cells did not increase uniformly. TaqMan-based real-time PCR analysis of mitochondrial transcripts showed that the mRNA levels of NADH dehydrogenase subunit 3, 4/4L, and 5 (*ND3*, *ND4/4L, and ND5*) were increased relative to control cells, whereas the mRNA levels of cytochrome c oxidase (COX) subunit I and III (*COXI, COXIII*), ATP synthase F0 subunit 6 and 8 (*ATP6/8*), and cytochrome b (*CytB*) were not changed (Fig. S1A). Moreover, northern blot analysis showed the accumulation of longer RNAs, which are likely unprocessed bicistronic *COXIII*-*ATP6/8* and *ND6-CytB* mRNAs respectively, in ClpP knockdown cells (Fig. 1C). Accumulation of unprocessed transcripts was confirmed using RT-PCR (Fig. S1B). Therefore, the increase of ND6 mRNA by RT-qPCR analysis was almost due to the increase of unprocessed RNA in ClpP knockdown cells (Figs 1B and S1A). While, mitochondrial protein synthesis was reduced modestly (Fig. 1F).

### ClpXP regulates the levels of mitochondrial RNA binding proteins

**Figure 2:**
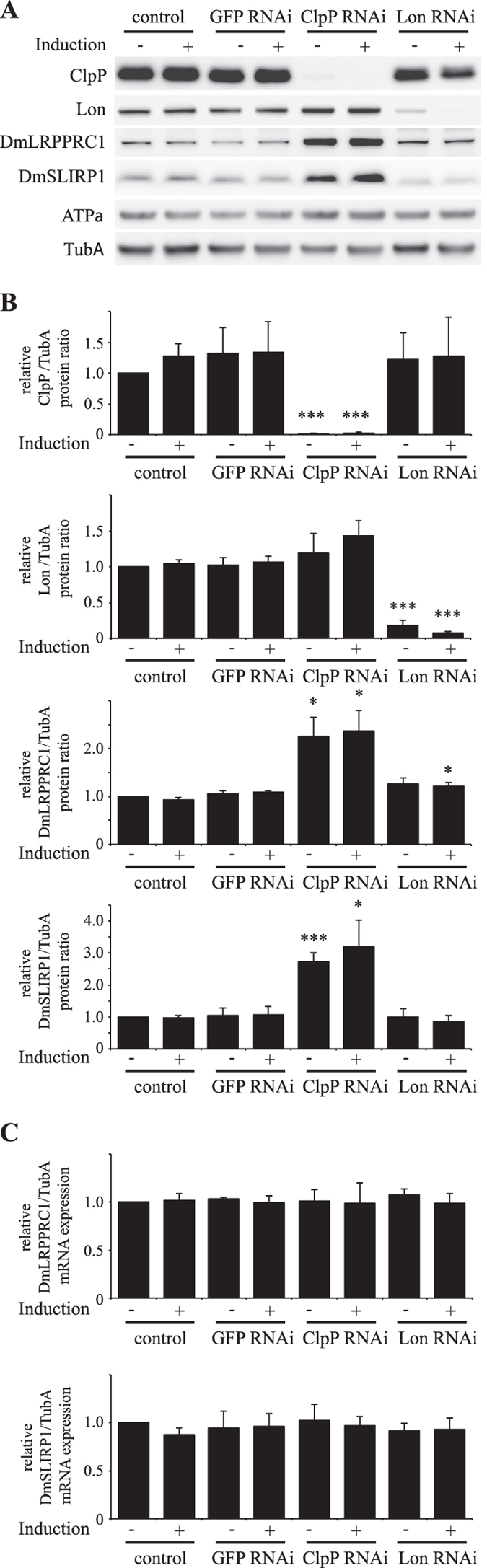
Protein levels of DmLRPPRC1 and DmSLIRP1 in ClpP-depleted Schneider S2 cells. Schneider S2 cells with no plasmid (control) or carrying pMt/invGFP/Hy (GFP RNAi) or pMt/invClpP/Hy (ClpP RNAi) or pMt/invLon/Hy (Lon RNAi) were cultured for 5 d in the presence or absence of 0.1 mM CuSO4. (**A**) Immunoblot analysis was carried out as described in the legend to Fig. 1A. (**B**) The relative protein levels of ratios ClpP, Lon, DmLRPPRC1 or DmSLIRP1. The ratios were normalized as described in Fig. 1B. Data represent the mean ± SD of four independent experiments. *P < 0.05; ***P < 0.001 versus control cells without induction (Student’s t-test). (**C**) The relative ratio of DmLRPPRC1 and DmSLIRP1 mRNA. The ratio was determined as described in Fig. 1D. Data represent the mean ± SD of three independent experiments. Full blots are presented in Supplementary Fig. S9.

Despite the mRNAs of *ND3 COXIII*, *ATP6/8*, *COXII* and *COXI* being generated from the same polycistronic transcripts (Fig. S1B), ClpP depletion alters the levels of these mRNAs non-uniformly. Therefore, we hypothesized that this results from differential alteration of mRNA stability. We examined the levels of the mitochondrial RNA binding proteins DmLRPPRC1 and DmSLIRP1 (CG33714). The former affects mt-mRNA stability and the latter is supposed to be a DmLRPPRC1-associated protein^26^. ClpP depletion increased DmLRPPRC1 and DmSLIRP1 proteins more than two times, whereas depletion of mitochondrial Lon protease caused only a slight increase in their accumulation (Fig. 2A,B). To assess whether the increase in DmLRPPRC1 and DmSLIRP1 is due to an increase of their associated mRNAs, their mRNA levels were quantified by RT-qPCR. Neither was changed significantly in ClpP knockdown cells (Fig. 2C). To exclude the possibility of off-target effect of RNAi, we established another RNAi cell line that contains different, non-overlap target region. The protein levels of DmLRPPRC1 and DmSLIRP1 were also increased in this cell line (Fig. S2). Transient suppression of ClpP with dsRNA also results in an increase of DmLRPPRC1 and DmSLIRP1 proteins in Schneider S2 cells (Fig. S3), suggesting that the increase of DmLRPPRC1 and DmSLIRP1is not due to adaptive changes during cell cloning.

Because ClpP forms a barrel-shaped, hetero-oligomeric complex with the chaperone-like subunit ClpX (CG4538), we next established ClpX knockdown cell lines and measured the protein levels of DmLRPPRC1 and DmSLIRP1. Schneider S2 cells stably expressing pMt/invClpX/Hy were cultured for 5 d in the absence or presence of 0.1 mM CuSO_4_, and the cells expressed >8-fold less ClpX than control cells (Fig. S4A,B). Similar to ClpP knockdown cells, knockdown of ClpX also resulted in an increase of DmLRPPRC1 and DmSLIRP1 protein without an increase in their corresponding mRNAs (Fig. S4A–C). Taken together, our results show that ClpXP regulates the protein levels of DmLRPPRC1 and DmSLIRP1.

### ClpXP specifically degrades DmLRPPRC1, not DmSLIRP1

**Figure 3:**
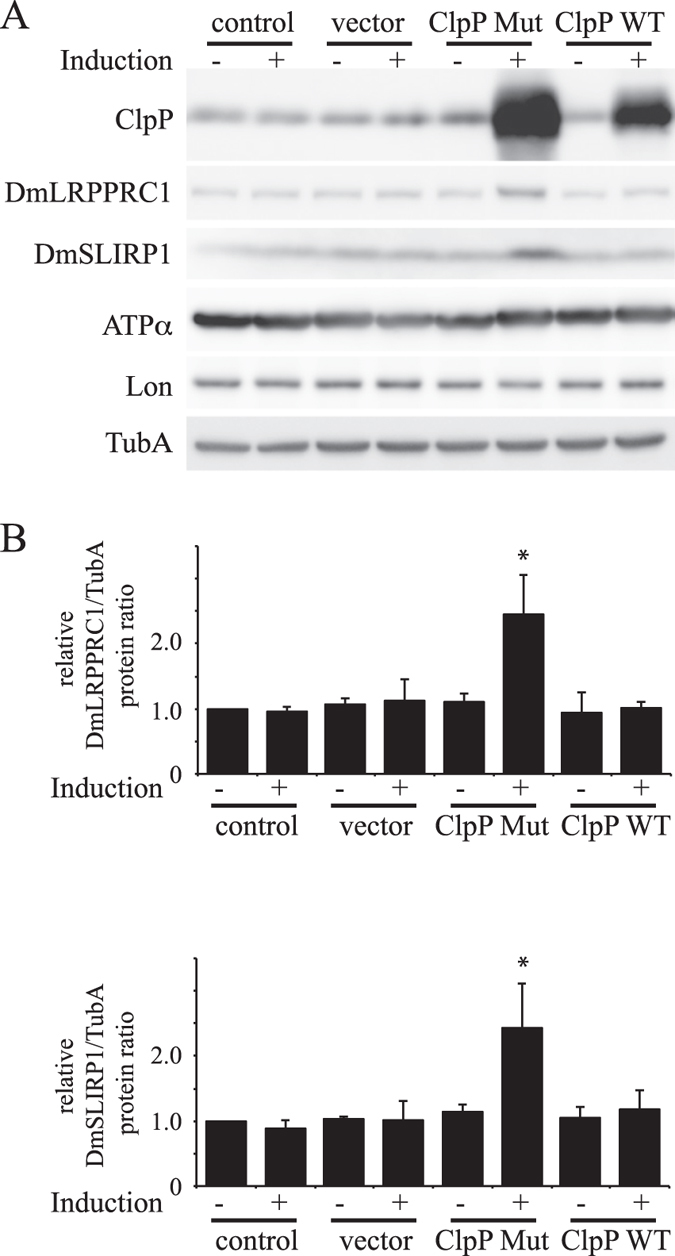
Protein levels of DmLRPPRC1 and DmSLIRP1 in ClpP mutant-overexpressing Schneider S2 cells. Schneider S2 cells carrying pMt/ClpP WT/Hy (ClpP WT), pMt/ClpP S124A/Hy (ClpP Mut) were cultured for 5 d. (**A**) Immunoblot analysis was carried out as described in the legend to Fig. 1A. (**B**) The relative ratio of DmLRPPRC1 and DmSLIRP1 protein. The ratio was determined as described in Fig. 1B. The results represent the mean ± SD of three independent experiments and *P < 0.05 versus control cells without induction. Full blots are presented in Supplementary Fig. S9.

To determine if ClpXP-mediated proteolysis regulates the protein levels of DmLRPPRC1 and DmSLIRP1, we established a copper-inducible cell line expressing a catalytically inactive ClpP mutant (S124A) in which the conserved serine in the proteolytic active site was replaced with alanine. Immunoblot analysis of copper-treated cells showed a 20-fold increase in the level of ClpP in cells carrying the S124A substitution relative to control cells (Fig. 3A). The cell line expressing ClpP S124A exhibited increased amounts of DmLRPPRC1 and DmSLIRP1, likely because overexpression of ClpP S124A results in a dominant negative phenotype that is caused by the formation of mixed oligomeric forms (Fig. 3A,B). On the other hand, cells expressing wild-type ClpP did not show significant changes in either protein (Fig. 3A,B). These results suggest ClpXP degrades DmLRPPRC1 and/or DmSLIRP1.

**Figure 4:**
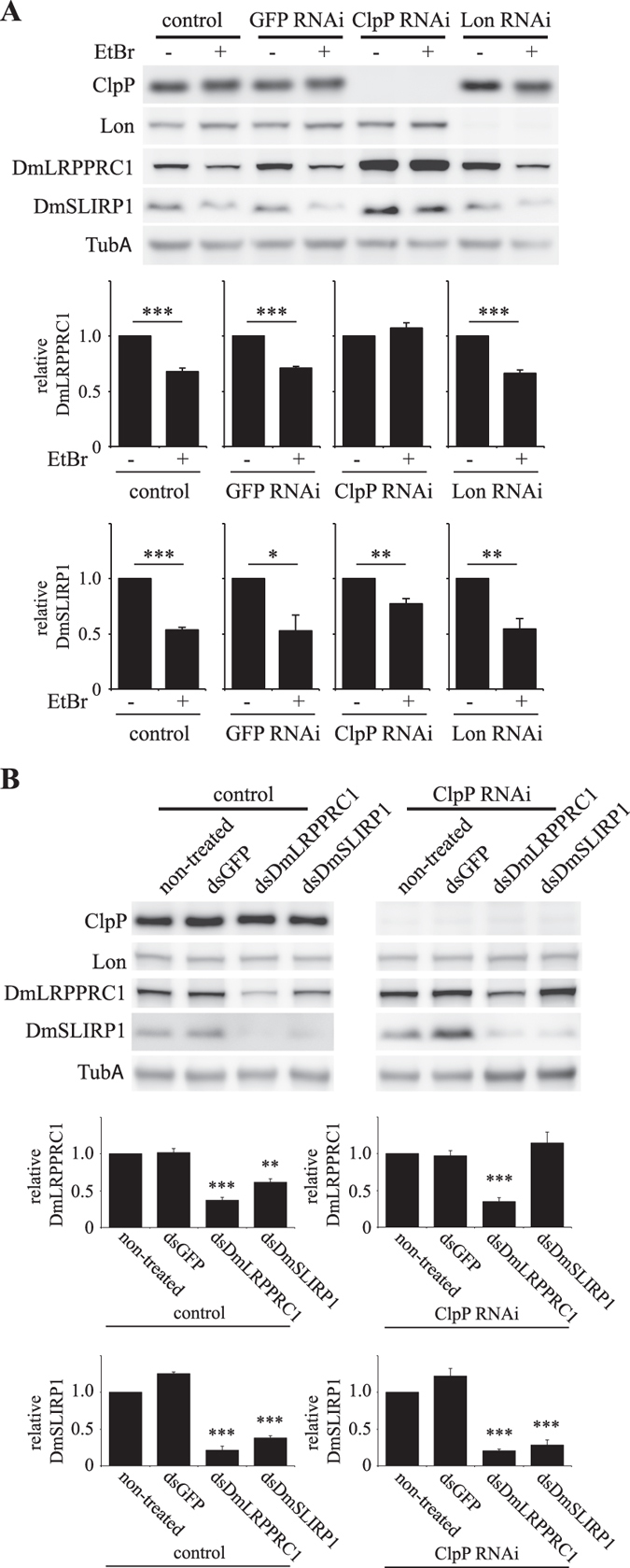
Dynamics of DmLRPPRC1 and DmSLIRP1 proteins during mitochondrial transcript depletion in ClpP or Lon knockdown Schneider S2 cells. (**A**) Schneider S2 cells with no plasmid (control) or carrying pMt/invGFP/Hy (GFP RNAi) or pMt/invClpP/Hy (ClpP RNAi) or pMt/invLon/Hy (Lon RNAi) were cultured for 2 d in the presence or absence of 200 ng∕mL EtBr. *Upper panel*, immunoblot analysis was carried out as described in the legend to Fig. 1A. *Lower panel*, reduction ratio of DmLRPPRC1 and DmSLIRP1 protein with or without EtBr treatment. Relative ratio of each cell lines was determined as described in Fig. 1B and normalized by the ratios of each cells without EtBr treatment. The results represent the mean ± SD of three independent experiments. *P < 0.05; **P < 0.01; ***P < 0.001 versus each untreated cells. (**B**) Schneider S2 cells carrying no plasmid (control) and or pMt/invClpP/Hy (ClpP RNAi) were cultured for 8 d in the absence (non-treated) or presence of dsRNA of DmLRPPRC1 (dsLRPPRC1), DmSLIRP1 (dsSLIRP1) or GFP (dsGFP) as control. *Upper panel*, immunoblot analysis was carried out as described in the legend to Fig. 1A. *Lower panel*, the relative DmLRPPRC1 or DmSLIRP1 protein ratio with or without dsRNA treatment. Relative ratio of each cell lines was determined as described in Fig. 1B and normalized by the ratios of each cells without dsRNA treatment. **P < 0.01; ***P < 0.001 versus each untreated cells. Full blots are presented in Supplementary Fig. S9.

Because it has been confirmed that reduction of mitochondrial transcription causes destabilization of LRPPRC and SLIRP in human cells^30, 31^, we tested this phenomenon in *Drosophila* Schneider S2 cells. To decrease mitochondrial transcription, we cultured the cell lines in the presence of ethidium bromide (EtBr), an inhibitor of mitochondrial transcription (Fig. 4A). EtBr treatment decreased the protein levels of DmLRPPRC1 and DmSLIRP1 in control cells (lanes 1–4). EtBr treatment reduced DmLRPPRC1 protein even in Lon RNAi cells (lanes 7 and 8). In contrast, the relative protein level of DmLRPPRC1 was unchanged in ClpP RNAi cells in the presence of EtBr (lanes 5 and 6), suggesting that DmLRPPRC1 is degraded by ClpXP. In contrast, DmSLIRP1 was reduced both in control and ClpP RNAi cells (lanes 5 and 6), which indicates that the EtBr-induced degradation of DmSLIRP1 does not depend on ClpXP.

It has been shown that stability of LRPPRC and SLIRP is interdependent in human cells^27, 30, 31^. The same interdependency was also observed in Schneider S2 cells as 8 d knockdown of DmLRPPRC1 and DmSLIRP1 resulted in a reduction of DmSLIRP1 and DmLRPPRC1 protein in control cells, respectively (Fig. 4B, left panels). In ClpP knockdown cells, knockdown of DmLRPPRC1 reduced DmSLIRP1, but DmSLIRP1 knockdown did not reduce DmLRPPRC1 (Fig. 4B, right panel) as EtBr did not (Fig. 4A). Moreover, to examine the stability of DmLRPPRC1 protein in ClpP knockdown cells, we monitored DmLRPPRC1 protein levels after addition of cytosolic protein synthesis inhibitors. In control cells, the levels of DmLRPPRC1 were decreased over time, whereas the levels of DmLRPPRC1 were stabilized in ClpP knockdown cells (Fig. S5). On the other hand, the protein levels of DmSLIRP1 were decreased over time in both cell limes. These results further strengthen the idea that ClpXP degrades specifically DmLRPPRC1.

**Figure 5:**
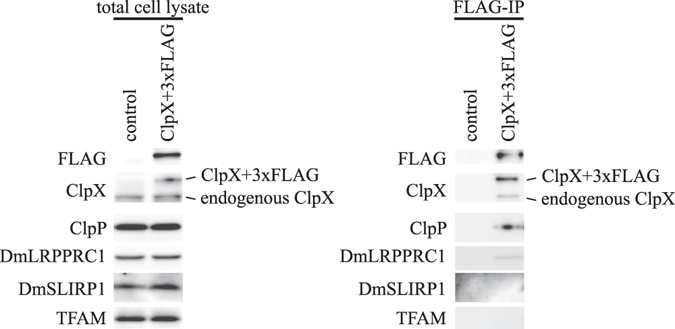
DmLRPPRC1 forms a complex with ClpXP. pMt/ClpX-FLAG/Hy was transfected into Schneider S2 Cells expressing a catalytically inactive ClpP mutant (S124A) and FLAG-tagged ClpX protein was immunoprecipitated using anti-FLAG-agarose. Total cell lysates (*left*) and immumoprecipitated proteins (*right*) were analysed by western blotting using specific antibodies. The results are representative of two independent experiments. Full blots are presented in Supplementary Fig. S9.

To see direct interaction between ClpXP and DmLRPPRC1, we performed immunoprecipitation analysis with FLAG-tagged ClpX protein. As shown in Fig. 5, FLAG-tagged ClpX is co-immunoprecipitated with ClpP and DmLRPPRC1 but not DmSLIRP1. Thus, ClpXP makes physical association with DmLRPPRC1 in Schneider S2 cells.

Our results show that ClpP regulates DmLRPPRC1 levels in *Drosophila* cells. A recent report indicated that knockdown of Lon protease inhibits LRPPRC degradation in mammalian cells. However whether or not ClpXP is involved in the degradation of LRPPRC was not evaluated. To evaluate this, we cultured CLPP or LONP1 knockdown HeLa cells in the presence of ethidium bromide (Fig. S6), using a protocol similar to that in the experiment shown in Fig. 4A. In the control cells, EtBr treatment resulted in a severe reduction in the LRPPRC protein (Fig. S6). In comparison with the control, the decrease in LRPPRC protein was suppressed moderately in the LONP1 knock down cells (Fig. S6). Similar to the case in the LONP1 knockdown cells, the decrease in LRPPRC protein was also suppressed moderately in the CLPP knock down cells (Fig. S6). Interestingly, knockdown of LONP1 or CLPP does not suppress the degradation of LRPPRC protein entirely (Fig. S6). These results suggest that in addition to LONP1, ClpXP may also contribute to the degradation of LRPPRC protein in mammalian cells.

### DmLRPPRC1 mediates effects of ClpXP knockdown

**Figure 6:**
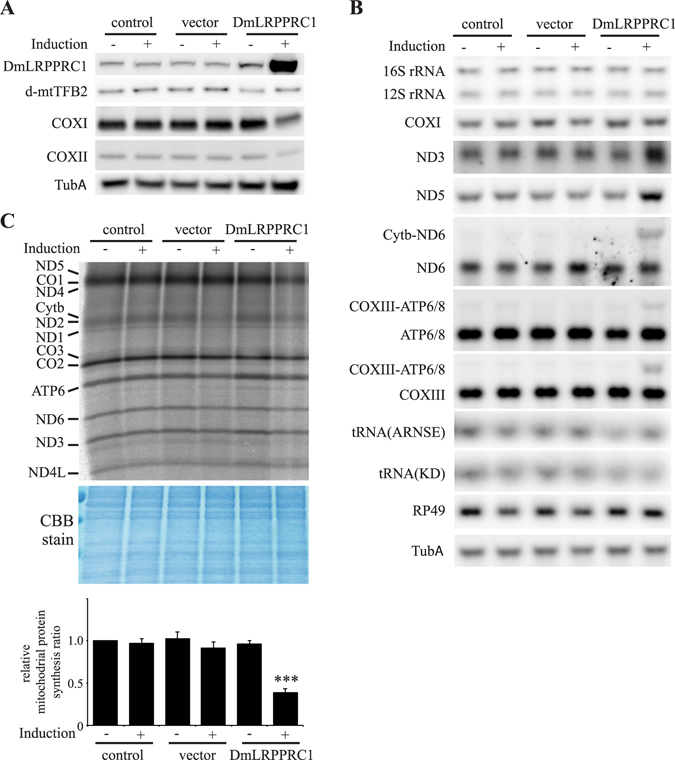
Effects of the protein levels of DmLRPPRC1 on mitochondrial protein synthesis in Schneider S2 cells. (**A**) Schneider S2 cells with no plasmid (control) or carrying pMt/Hy (vector) or pMt/DmLRPPRC1/Hy (DmLRPPRC1) were cultured for 5 d in the presence or absence of 0.1 mM CuSO_4_. Immunoblot analysis was carried out as described in the legend to Fig. 1A. (**B**) Steady-state levels of mitochondrial transcripts in DmLRPPRC1-overexpressing Schneider S2 cells. Northern blot analysis of the levels of mitochondrial genome encoded transcripts in ClpP-depleted Schneider S2 cells. The nuclear genome encoded transcripts, *RP49 and TubA*, was used as a loading control. (**C**) Mitochondrial translation products were labeled in Schneider S2 cells after 5 d of culture in the presence or absence of 0.1 mM CuSO_4_. Labeling was carried as described in Fig. 1F. *Upper panel*, aliquots containing 10 μg of total protein each were fractionated by 15–20% gradient SDS-PAGE. Coomassie Brilliant Blue (CBB) staining was used to check for equal loading of the samples. *Lower panel*, the relative mitochondrial protein synthesis ratio of DmLRPPRC1-overexpressing cells. The relative mitochondrial protein synthesis ratio was quantitated as described in Fig. 1F. The results represent the mean ± SD of three independent experiments and ***P < 0.001 versus control cells without induction (Student’s t-test). Full blots are presented in Supplementary Fig. S9.

To see the functional significance of increased DmLRPPRC1 caused by ClpXP depletion, we established copper-inducible cell lines capable of overexpressing DmLRPPRC1. After 5 d of incubation in the presence of copper, immunoblot analysis indicated an 8-fold increase in DmLRPPRC1 relative to control cells (Fig. 6A). The effect of DmLRPPRC1 overexpression on mitochondrial transcript abundance was evaluated by northern blots and RT-qPCR (Figs 6B and S7). The levels of *ND3*, *ND4/4L* and *ND5* mRNAs increased in cells that overexpressed DmLRPPRC1 (Figs 6B and S7). However, the mRNA levels of *COXI*, *COXIII*, *ATP6/8*, and *CytB* were not significantly changed (Figs 6B and S7). Furthermore, DmLRPPRC1-overexpressing cells also showed an increase in unprocessed *COXIII*-*ATP6/8* and *ND6*-*CytB* mRNAs (Fig. 6B). Thus, overexpression of DmLRPPRC1 does not uniformly increase mt-mRNAs, similar to ClpP knockdown, which suggests the phenomena observed in ClpP knockdown cells are mediated by DmLRPPRC1.

We did not detect an increase in mitochondrial rRNAs or tRNAs (Fig. 6B). In addition, the protein levels of mtTFB2 were not changed in DmLRPPRC1-overexpressing cells (Fig. 6A). These indicate that the increased protein levels of DmLRPPRC1 do not affect mitochondrial transcription in Schneider S2 cells.

### Excess DmLRPPRC1 reduces the efficiency of mitochondrial translation

DmLRPPRC1 overexpression did not reduce the levels of mtDNA-encoded mRNAs, whereas the levels of the mtDNA-encoded proteins COXI and COXII were severely reduced (Fig. 6A). To evaluate the synthesis of mitochondrial proteins in DmLRPPRC1-overexpressing cells, we performed pulse labeling of mitochondrial translation, and found that mitochondrial protein synthesis ratio in DmLRPPRC1-overexpressing cells was decreased to 40% of that in control cells. (Fig. 6C). Excess DmLRPPRC1 may interfere with mitochondrial translation in Schneider S2 cells.

**Figure 7:**
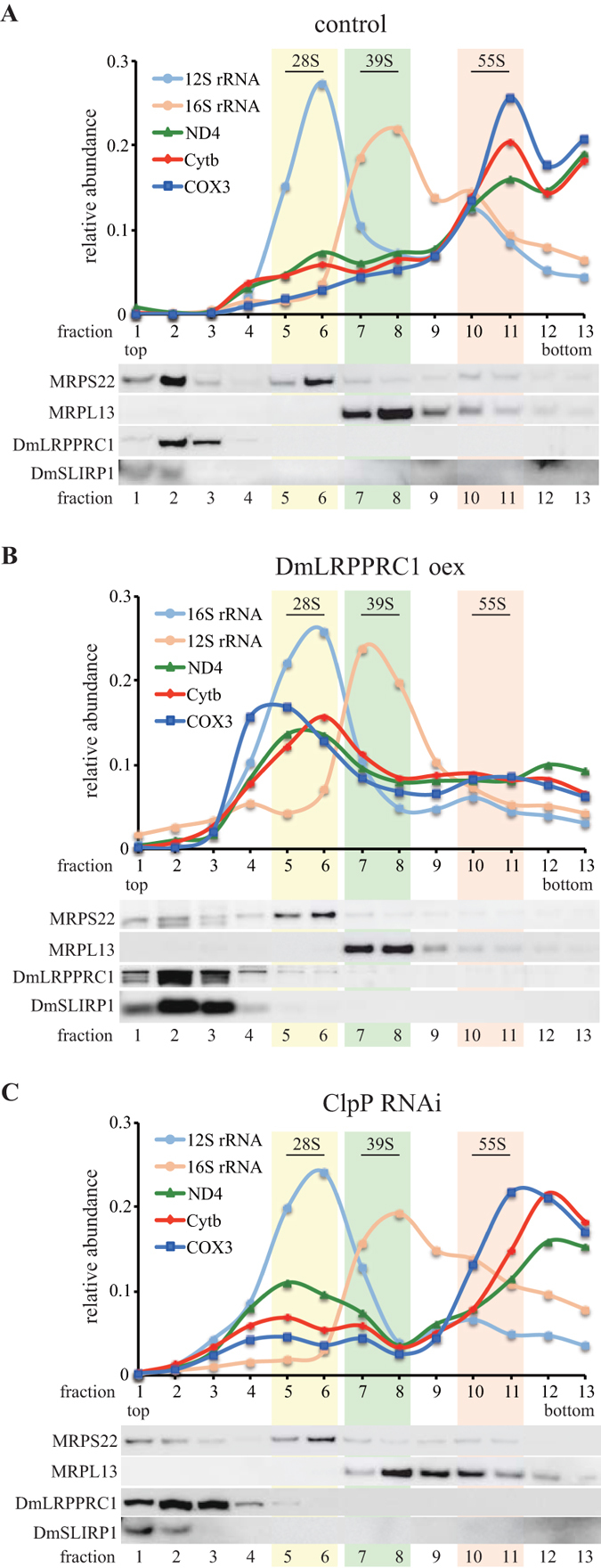
Effects of the protein level of DmLRPPRC1 on mitochondrial ribosome assembly. An increase in DmLRPPRC1 expression leads to impaired assembly of mitoribosomes. Sedimentation analysis of mitoribosomal particles by sucrose gradient centrifugation in Schneider S2 cells (**A**), DmLRPPRC1 overexpressing cells (**B**) and ClpP knockdown cells (**C**). Individual mitochondrial transcripts were analyzed by TaqMan-based real-time PCR assays. *Upper panel*, the abundance of mitochondrial transcripts in each fraction is shown as the ratio of the sum of the abundance in all fractions. *Lower panel*, the migration of the small (28S) and the large (39S) ribosomal subunits and the monosome (55S) is assessed using subunit specific antibodies and is indicated for reference. Full blots are presented in Supplementary Fig. S9.

We further investigated the reduced mitochondrial translation in DmLRPPRC1-overexpressing cells by performing sucrose gradient sedimentation analysis of mitochondrial extracts. Mitochondrial extracts were prepared from control cells, DmLRPPRC1-overexpressing cells and ClpP knockdown cells. As shown in the ClpP deficient mouse, mitochondrial ribosomal proteins accumulated (Fig. S8). To determine the sedimentation profile of small ribosomal subunits (28S), large ribosomal subunits (39S) and assembled ribosomes (55S), we measured the levels of mitochondrial ribosomal proteins and mitochondrial ribosomal RNAs by immunoblot and RT-qPCR, respectively. In the DmLRPPRC1 overexpressing cells the relative amount of mt-mRNAs in the assembled ribosome fraction was severely reduced compared to the control cells. This was accompanied by an increase in mt-mRNAs in fractions 4–6, which corresponds closely to fractions containing the small ribosomal subunit (fractions 5–6) (Fig. 7A,B). These results indicate that excess DmLRPPRC1 causes mitochondrial translation defects by preventing ribosome assembly. Similar to DmLRPPRC1 overexpressing cells, the protein levels of DmLRPPRC1 are increased (Fig. 2) and mitochondrial translation was reduced modestly (Fig. 1) in ClpP knockdown cells. Interestingly, in ClpXP knockdown cells, sucrose gradient sedimentation showed a modest reduction of mt-mRNAs in assembled ribosomes (55S) fractions, and a moderate increase in mt-mRNAs in small ribosomal subunit fractions (fractions 4–6), analogous to that which we observed in DmLRPPRC1-overexpressing cells (Fig. 7B,C). Taken together, in addition to regulation of mt-mRNA processing and stability, our data show that ClpXP also modulates mitochondrial translation via regulating the accumulation of DmLRPPRC1.

## Discussion

Mitochondrial transcripts were increased in ClpP knockdown cells (Fig. 1C), which may result from upregulation of mitochondrial transcription activity because mitochondrial tRNAs, which can be indicators of mitochondrial transcription activity, were increased in addition to upregulation of mtTFB2 protein and mRNA (Fig. 1A,B,D). We showed previously that mitochondrial transcripts are also increased in Lon knockdown cells in association with an increase in mtTFB2 protein. These similar events might suggest that upregulation of mtTFB2 is a common response to defects in mitochondrial matrix protein turnover caused by depletion of either Lon or ClpXP. Moreover, mtDNA copy number is also increased in ClpP knockdown cells. These results might suggest that the increase in mtDNA copy number results from the increased levels of mitochondrial transcripts that are required for the synthesis of RNA primers during replication of mtDNA, as we reported previously^33^. Another possible explanation is that the increase in mtDNA is caused by accumulation of other mtDNA replication factors by either direct or indirect effects of ClpP knockdown.

DmLRPPRC1 overexpression did not change the abundance of mitochondrial tRNAs (Fig. 6B). Therefore, DmLRPPRC1 may not be involved in mitochondrial transcription, which is in agreement with previous studies showing no link between LRPPRC and transcriptional activity^36^. Bratic et. al. showed that depletion of DmLRPPRC1 results in the reduction of mitochondrial mRNA levels^26^ and the same phenomenon was observed also in vertebrate cells^26, 27^. In addition, inhibition of mitochondrial transcription reduces LRPPRC and SLIRP levels in vertebrate cells^29, 31^. These results indicate that levels of LRPPRC, SLIRP and mt-mRNAs are interdependent. Likewise, we showed here that DmLRPPRC1 degradation is facilitated in some conditions, such as inhibition of mitochondrial transcription or depletion of DmSLIRP1 in *Drosophila* cells (Fig. 4). Conversely, DmLRPPRC1 increased when ClpP or ClpX was knocked down (Fig. 2A lanes 5, 6 and Fig. S4A lanes 5, 6), suggesting that DmLRPPRC1 is degraded by ClpXP. In agreement with this hypothesis, ClpP depletion prevented the facilitated degradation of DmLRPPRC1 caused by mitochondrial transcription inhibition (Fig. 4A, compare lanes 5 and 6) or DmSLIRP1 knockdown (Fig. 4B, compare lanes 4 and 8). DmLRPPRC1 may be specifically degraded by ClpXP because Lon-depletion did not accumulate DmLRPPRC1 (Fig. 2A lanes 7, 8) or inhibit the facilitated degradation of DmLRPPRC1 (Fig. 4A, compare lanes 7 and 8). Moreover, we showed that ClpX, which recognize the protein substrates of ClpXP, physically interacts with DmLRPPRC1 in the cells (Fig. 5). Collectively, our results strongly suggest that DmLRPPRC1 is a specific substrate of ClpXP in *Drosophila* cells. By contrast, ClpP knockdown did not prevent the degradation of DmSLIRP1 caused by mitochondrial transcription inhibition (Fig. 4A, compare lanes 5 and 6) or DmLRPPRC1 knockdown (Fig. 4B, compare lanes 3 and 7), which suggests that the facilitated degradation of DmSLIRP1 is not specifically mediated by ClpXP. DmLRPPRC1 and DmSLIRP1 stabilize each other. It is considered that ClpP knockdown increased DmSLIRP1 protein in control cells (Fig. 2A lanes 5, 6) due to the DmLRPPRC1 accumulation.

The preferred amino acid sequences recognized by mitochondrial ClpXP remain unclear. At the same time, proteomic studies of *E. coli* ClpXP identified some classes of peptide sequences, called preferred degradation tags, and most of the preferred C-terminal degradation tags contain alanine in the C-terminal position^6^. Interestingly, the amino acid in the C-terminal position of DmLRPPRC1 is alanine, suggesting that mitochondrial ClpXP might also recognize the alanine. Our results show the possibility that in addition to LONP1, ClpXP may also contribute to the degradation of LRPPRC protein. However, unlike the situation in Schneider S2 cells, ClpXP is not the dominant protease in the degradation of LRPPRC protein in HeLa cells. Moreover, a recent report indicated that knockdown of Lon protease inhibits LRPPRC degradation in mouse cells^30^. Because the C-terminal amino acid in mouse and human LRPPRC is not alanine, these differences in the substrate preference of proteases between species might arise from differential recognition of C-terminal degradation tags.

A recent paper showed that overexpressing ClpX in mouse C2C12 cells results in an increase in LRPPRC protein possibly involving the mitochondrial unfolded protein response pathway^18^. This indicates that changes in the protein levels of ClpX may affect the gene expression of LRPPRC in mammalian cells. Here, we showed that knockdown of ClpX or ClpP results in an increase of DmLRPPRC1 protein without an increase in the corresponding mRNA (Figs 2 and S4). These data indicate that in ClpP or ClpX knockdown Schneider S2 cells, the increase of DmLRPPRC1 likely does not result from signal transduction events.

Our study showed that two unprocessed mRNAs, *COXIII-ATP6/8* and *ND6-CytB*, are accumulated in ClpP knockdown cells (Fig. 1C) or DmLRPPRC1 overexpression cells (Fig. 6B). Metazoan mtDNA is transcribed as precursor polycistronic transcripts containing mt-mRNAs, mt-rRNAs and mt-tRNAs. Most of the mt-mRNAs and mt-rRNAs are produced when mt-tRNAs are excised by ELAC2 and the RNaseP-complex^21, 22^. However, some processing sites are cleaved without an accompanying mt-tRNA excision, such as *COXIII-ATP6/8* and *ND6-CytB* in *Drosophila*. The mechanisms involved in these cleavages remain largely unknown. In human cells, the ELAC2 and RNaseP-complexes do not appear to be involved in non-tRNA processing. Recent studies suggest that pentatricopeptide repeat domain 2 protein, which has one PPR domain, contributes to non-tRNA cleavages^37, 38^. DmLRPPRC1 contains multiple PPR domains. Excess DmLRPPRC1 protein may compete with canonical proteins performing the non-tRNA processing of *COXIII-ATP6/8* and *ND6-CytB*.

An increase of DmLRPPRC1 protein results in a non-uniform increase in mitochondrial mRNA expression. Similar to the previously reported functions of vertebrate LRPPRC, DmLRPPRC1 is responsible for polyadenylation of mitochondrial mRNAs, which affects the stability of mt-mRNAs^26^. However, we did not detect any difference in migration of mt-mRNAs in northern blot analysis in ClpP RNAi cells (Fig. 1C) and DmLRPPRC1 overexpressing cells (Fig. 6B). These results suggest that the length of poly(A) tails of mt-mRNAs are not changed dramatically in the presence of excess DmLRPPRC1 protein.

We showed that excess DmLRPPRC1 resulted in a reduction of mitochondrial translation. These results are consistent with the finding of Bratic *et al*.^26^ who showed that depletion of DmLRPPRC1 causes an increase in mitochondrial translation in flies. Similar to our results, in *Schizosaccharomyces pombe*, overexpression of a multiple PPR domain-containing protein, PPR5, resulted in inhibition of mitochondrial protein synthesis without an obvious change in steady state levels of mitochondrial transcripts^39, 40^. Mitochondrial protein synthesis is reduced more severely in DmLRPPRC1 overexpressing cells although levels of mitochondrial mRNAs do not differ between ClpP knockdown cells and DmLRPPRC1 overexpression cells. DmLRPPRC1 was increased about two-fold in the former cells while about eight-fold in the latter. We showed previously that the ratio of TFAM to mtDNA is important for the regulation of mitochondrial transcription and a higher ratio of TFAM to mtDNA causes a reduction in mitochondrial transcript abundance^5, 17^. Analogously, the ratio of DmLRPPRC1 to mtRNAs may be critical for the regulation of mitochondrial translation.

Our ribosome profiling (Fig. 7) results showed that DmLRPPRC1 overexpression inhibited the formation of fully assembled 55S ribosomes, and enhanced the accumulation of mt-mRNAs in the lower-density fractions that contain mitochondrial small ribosomal subunits. In mitochondrial translation, mt-mRNAs initially interact with the small ribosomal subunit and then are assembled into the full ribosome^41, 42^. Therefore, our results suggest that excess DmLRPPRC1 does not prevent this interaction, but instead interferes with the assembly of mt-mRNA-small ribosomal subunit complexes with large ribosomal subunits.

In humans, recessive mutations of ClpP have been known to cause Perrault syndrome characterized by sensorineural hearing loss and ovarian failure^10^. Similar to humans, ClpP knockout mice show hearing loss and infertility^12^. Interestingly, knockout of ClpP also causes ineffective mitochondrial translation^43^. A very recent study shows that ClpXP regulates the protein levels of ERAL1^44, 45^, a putative 12S rRNA chaperone. ERAL1 protein was accumulated in ClpP deficient mouse heart and the excess ERAL1 cause ineffective mitochondrial translation by the inhibition of mitochondrial ribosome assembly^43^. To date, many proteins are on the list of candidates of ClpXP substrates^16, 43, 46, 47^. Therefore, some substrate proteins of ClpXP may be involved in mitochondrial translation. Further studies are necessary to clarify the relationship between protein substrates of ClpXP and mitochondrial translation.

In summary, we conclude that DmLRPPRC1 is a specific substrate of ClpXP. An increase in DmLRPPRC1 protein, which can result from ClpXP depletion, causes non-uniform increases of mitochondrial mRNAs, accumulation of some unprocessed mitochondrial transcripts and partial inhibition of mitochondrial translation.

## Experimental Procedures

Cloning and molecular biology - The plasmids pMt/invClpP/Hy and pMt/invLon/Hy which carry an inverted repeat of a nucleotide sequence from cDNA, were constructed in the same way as described previously^17, 32–34^ with the primers listed in Supporting Table S1. The plasmids pMt/ClpP WT/Hy and pMt/ClpP S124A/Hy in which ClpP is regulated by the metallothionein promoter, were constructed in the same way as described previously^34^ with the primers listed in Supporting Table S1. The plasmid pMt/invLon/Hy was constructed as described previously^17^.

Generation and induction of stable cell lines - *Drosophila* Schneider S2 cells were cultured at 25 °C in *Drosophila* Schneider Medium (Invitrogen) supplemented with 10% FBS. Cells were subcultured to 3 × 10^6^ cells∕mL every fourth day. Cells were transfected using Effecten (QIAGEN). Hygromycin-resistant cells were selected with 200 μg∕mL hygromycin. Cells were cultured for 8 w in hygromycin-containing medium and then passaged in standard medium. We established two or more independent lines for each construct and analysed. The cell lines were grown to a density of 3 × 10^6^ cells ∕mL and then treated with 0.1 mM CuSO4 to induce expression from the metallothionein promoter. The experiments shown here were performed more than three times with each of the two or three independent cell lines carrying each plasmid construct, including control (no plasmid).

Preparation of antibodies - Antibodies against DmLRPPRC1, DmSLIRP1, MRPS22, MRPL13, and ClpP, were prepared by immunizing rabbits with recombinant proteins of DmLRPPRC1 (aa 149–530), DmSLIRP1 (aa 26–90), MRPS22 (aa 42–397), MRPL13 (aa 43–178), and ClpP (aa 22–270), respectively. For generation of polyclonal antibodies against COXI, COXII, and ClpX, rabbits were immunized with synthetic peptides of COXI (aa 498–511), COXII (aa 170–183), or ClpX (aa 658–671) respectively.

Immunoblot - Total cellular protein (10 μg per lane) was fractionated by 4–12% SDS-PAGE and transferred to PVDF membrane (Bio-Rad). Membranes were pre-incubated for 1 h with Block One (Nacalai Tesqque), followed by incubation for 1 h with Can Get Signal solution 1 (Toyobo) containing primary antibodies. Filters were washed four times with PBS containing 0.1% Tween 20, incubated for 1 h with Can Get Signal solution 2 (Toyobo) containing horseradish peroxidase-conjugated anti-rabbit or mouse IgG (Bio-Rad), and washed with PBS containing 0.1% Tween 20. Protein bands were visualized using Western Lightning ECL Pro (PerkinElmer). ECL luminescence was quantified on an ImageQuant LAS 4000mini (GE Healthcare). Antibodies against *Drosophila* Lon^17^, mtTFB2^33^, VDAC (abcam) ATPase α (MBL), human LRPPRC^45^, human SLIRP (abcam), human ClpP (abcam), human LONP1^45^, β actin (MBL), and α tubulin (MBL) were prepared and used as described. The ratios of the signals for ClpP/α tubulin, Lon/α tubulin, DmLRPPRC1/α tubulin, DmSLIRP1/α tubulin or LRPPRC/α tubulin were used to estimate the relative protein levels.

Northern/Southern blotting - Total cellular RNA was extracted using RNAiso plus (Takara). RNA (10 μg per lane) was fractionated on a 2.0% agarose/formaldehyde gel with MOPS (Dojindo) buffer, and transferred onto Hybond-N + nylon membrane (GE Healthcare). Northern blot analysis using RNA probes was performed with AlkPhos direct (GE Healthcare) and then analyzed with a LAS-4000 (GE Healthcare). The signal for *RP49* and *TubA* was used for loading controls. Southern blotting was performed as described previously^17, 32–34^.

RNA interference (RNAi) with dsRNA - To generate double-stranded RNA (dsRNA) for RNAi, sequences directed against the protein to be silenced were amplified by PCR from each cDNA. Each primer used in the PCR contained a T7 RNA promoter followed by sequences specific for the targeted genes. The PCR primers are listed in Supporting Table S1. PCR products were used as templates for *in vitro* transcription using the T7 Megascript RNA kit (Thermo). 1 × 10^7^ Schneider S2 cells were plated into a T25 flask in 5 mL of SFX-insect medium (Thermo) containing 50 μg of dsRNA. The cells were collected after 4 d culture and dsRNA treatment was repeated. After 4 d following the second dsRNA treatment, the cells were harvested for the analysis.

Analysis of mitochondrial protein synthesis - Schneider S2 cell lines were grown in the presence or absence of 0.1 mM CuSO4 for 5 d. Cells were harvested at room temperature, washed twice with methionine-free Grace’s insect culture medium (Thermo), and resuspended at 3 × 10^6^ cells/ml in methionine-free Grace’s insect culture medium supplemented with 10% dialyzed FBS, 200 μg/ml emetine (Wako Pure Chemicals), and 100 μg/ml cycloheximide (Wako Pure Chemicals). Five minutes after cell resuspension, EXPRE^35^S^35^S protein labeling mix (PerkinElmer) was added to a final concentration of 300 μCi/ml, and the cells were incubated for 45 min at 25 °C. After incubation, the cells were diluted with 2 volumes of Schneider Medium and then washed twice with PBS. The cells were lysed in 10 mM Tris-HCl (pH 7.5), 1 mM EDTA, and 1% SDS. Total cellular protein (10 μg/lane) was fractionated by 15–20% gradient SDS-PAGE. The gels were stained with Coomassie Brilliant Blue and dried. The gels were analyzed using a BAS-2500 (GE Healthcare). The relative mitochondrial protein synthesis ratio was quantitated by average of the radiolabeled products of mtDNA encoded subunits, COX1 and COX2.

Cycloheximide chase assay – The cell lines were cultured with in the presence of 200 μg/ml emetine and 100 μg/ml cycloheximide, and the cells were isolated at different time points. Total cell extracts were analyzed by immunoblot as described above.

Real-time RT-PCR - Total RNA, extracted with RNeasy Mini Kit (QIAGEN) from the cells, was used as a template to synthesize cDNA using ReverTra Ace qPCR RT Master Mix with gDNA Remover (Toyobo). Real-time PCR was performed using THUNDERBIRD Probe qPCR Mix (Toyobo) and StepOnePlus Real-Time PCR System (Thermo), and PCR products were quantified fluorometrically using PrimeTime qPCR assays (IDT). Each sample was analyzed in duplicate and alpha tubulin mRNA (TubA) was used as a reference. We generated standard curves with serial dilutions for each qPCR primer set and calculated the relative abundance of transcripts using these linear curves. The PCR primers and 6-FAM/ZEN/IBFQ-labeled probes were listed in Supplementary Table S2.

Co-immunoprecipitation Analysis - Schneider S2 cells, which have pMt/ClpP S124A/Hy, were transfected with plasmid expressing FLAG-tagged ClpX and culture for 3 days in the presence of 0.1 mM CuSO_4_. Cells (1 × 10^7^) were washed twice in PBS and then extracted in 500 μl of extraction buffer (50 mM Tris-HCl pH7.4, 150 mM NaCl, 1 mM MgCl_2_, 1% TritonX-100, 50 μg/ml RNase A (Sigma), and protease inhibitor cocktail (Wako Pure Chemicals)) by pipetting vigorously. The extract was cleared by centrifugation at 20 000 g, 4 °C, for 15 min, and the supernatant was recovered and an aliquot of total cell lysate was subjected to Western blotting analysis. Immunoprecipitation was performed by rotating the tubes for 3 hours. The rest of the samples were incubated with anti-FLAG-agarose Beads (Sigma) for 3 hours at 4 °C. Then the beads were washed four times with lysis buffer. FLAG-tagged proteins were eluted with elution buffer (0.3 mg/ml FLAG peptides in wash buffer).

Sucrose gradient sedimentation - Mitochondria, isolated from 2 × 10^8^ Schneider S2 cells as described before, were lysed in lysis buffer (260 mM sucrose, 100 mM KCl, 20 mM MgCl2, 10 mM Tris-Cl [pH 7.5], 1% lauryl maltoside, 5 mM 3-Mercapto-1,2-propanediol, protease inhibitor cocktail without EDTA (Roche), RNase Inhibitor (Promega)) on ice for 20 min. Lysates were cleared prior to loading onto the sucrose density gradient by centrifugation at 10,000 × g for 45 min at 4 °C. Lysates were loaded on a 1 ml 10–30% discontinuous sucrose gradient (50 mM Tris-Cl, 100 mM KCl, 10 mM MgCl_2_) and centrifuged at 24,000 rpm for 15 h in a Beckman SW41-Ti rotor. After centrifugation, 13 fractions were collected from the top and used for further analysis. RNA isolation from these fractions was performed with RNAiso Blood (Takara) and Dr. GenTLE precipitation carrier (Takara). Half of the total amount of extracted RNA collected from sucrose gradient fractions was used as a template to synthesize cDNA using ReverTra Ace qPCR RT Master Mix with gDNA Remover (Toyobo). Real-time PCR was performed as described above.

Transfection of siRNA and ethidium bromide treatment - HeLa cells were cultured in Dulbecco’s modified eagle medium supplemented with 10% heat-inactivated fetal calf serum. Transfection of siRNAs was performed using Lipofectamine RNAiMAX (Thermo) according to the manufacturer’s instructions. Three days after the first siRNA transfection, cells were re-transfected. Six hours after the second siRNA transfection, the medium was replaced with new medium with or without 50 ng/ml ethidium bromide and cells were cultured for another 3 days. Total cell extracts were analyzed by immunoblot as described above. The following siRNAs were used: non-target control (SIC001, Sigma), LONP1#1(SASI_Hs01_00202289, Sigma), LONP1#2(SASI_Hs01_00202290, Sigma), CLPP#1(SASI_Hs01_00025717, Sigma) CLPP#2(SASI_Hs01_00025719, Sigma).

## Electronic supplementary material

Supplementary Information
