# Supplementary material for: Drosophila protease ClpXP specifically degrades DmLRPPRC1 controlling mitochondrial mRNA and translation
Source: Sci Rep. 2017 Aug 16;7:8315. doi: 10.1038/s41598-017-08088-6 (PMC5559520; doi:10.1038/s41598-017-08088-6)
Supplement: Supplementary file 1 — Supplementary Information [file 41598_2017_8088_MOESM1_ESM.pdf]

Supplementary Information

Drosophila protease ClpXP specifically degrades DmLRPPRC1 controlling mitochondrial mRNA and translation

Yuichi Matsushima[1, 2 †,*], Yuta Hirofuji[1,3, †], Masamune Aihara[1], Song Yue[1], Takeshi Uchiumi[1], Laurie S. Kaguni[2,*], Dongchon Kang[1,*]

From [1]Department of Clinical Chemistry and Laboratory Medicine, Graduate School of Medical Sciences, Kyushu University, 3-1-1 Maidashi, Higashi-ku, Fukuoka 812-8582, Japan: [2]Department of Biochemistry and Molecular Biology, and Center for Mitochondrial Science and Medicine, Michigan State University, East Lansing Michigan 48824-1319, USA : [3]Section of Pediatric Dentistry, Division of Oral Health, Growth and Development, Faculty of Dental Science, Kyushu University, 3-1-1 Maidashi, Higashi-ku, Fukuoka 812-8582, Japan

A

B

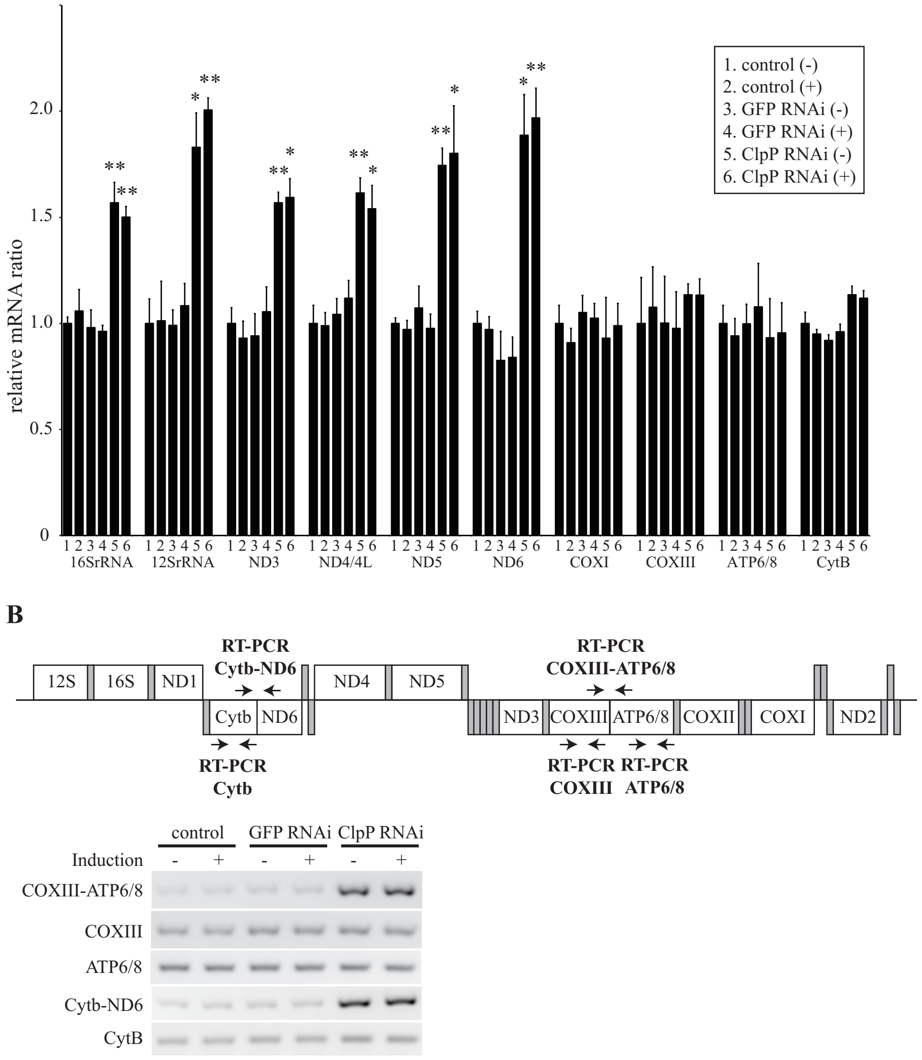

Figure S1. *Depletion of Drosophila* ClpP protease in Schneider cells. Schneider cells with no plasmid (control) or carrying pMt/invGFP/Hy (GFP RNAi) or pMt/invClpP/Hy (ClpP RNAi) were cultured for 5 d in the presence or absence of 0.1 mM CuSO4. (A) TaqMan-based real-time PCR assays of mitochondrial gene transcripts. The levels of the transcripts were standardized by nuclear encoded gene, α tubulin. Data were normalized to the expression level in control cells without induction for each RNA

species. The results represent the mean±SD of three independent experiments and *$P<0.05$; **$P<0.01$; versus control cells without induction. (B) Increase of unprocessed COXIII-ATP6/8 and Cytb-ND6 transcripts in ClpP-depleted Schneider cells. *Upper panel*, schematic gene map of the *Drosophila melanogaster* mitochondrial genome. Open boxes represent protein coding and ribosomal genes. Gray boxes are designated by tRNAs. The horizontal arrows represent the locations of PCR primers used for semi-quantitative RT-PCR analysis. *Lower panel*, total RNAs were analyzed by semi-quantitative RT-PCR. The PCR primers were listed in Table S2. PCR products were run on a 1.8% agarose gel and stained with ethidium bromide.

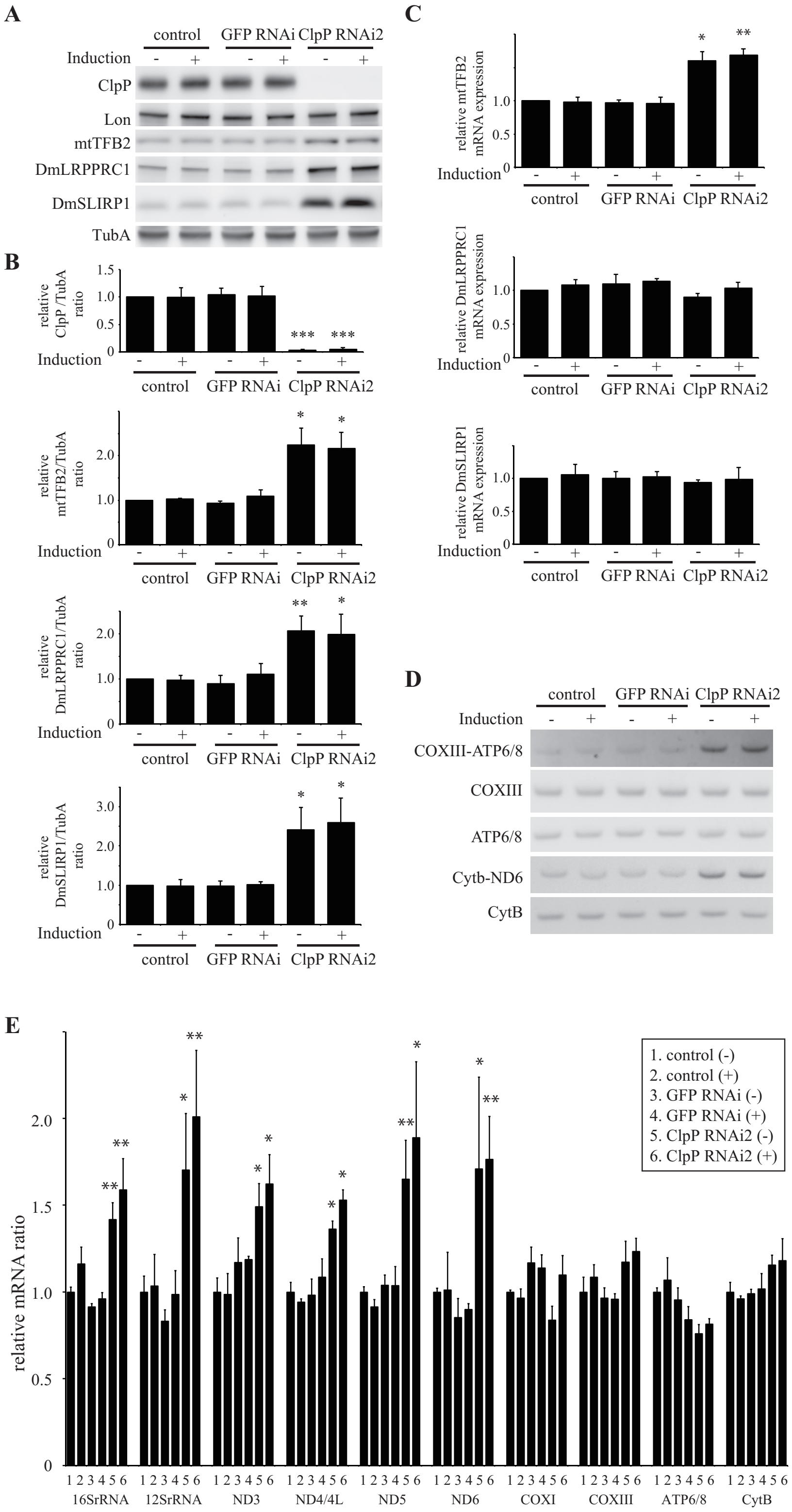
A
control
GFP RNAi
ClpP RNAi2
Induction
ClpP
Lon
mtTFB2
DmLRPPRC1
DmSLIRP1
TubA
B
relative ClpP/TubA ratio
relative mtTFB2/TubA ratio
relative DmLRPPRC1/TubA ratio
relative DmSLIRP1/TubA ratio
C
relative mtTFB2 mRNA expression
relative DmLRPPRC1 mRNA expression
relative DmSLIRP1 mRNA expression
D
COXIII-ATP6/8
COXIII
ATP6/8
Cytb-ND6
CytB
E
relative mRNA ratio
1. control (-)
2. control (+)
3. GFP RNAi (-)
4. GFP RNAi (+)
5. ClpP RNAi2 (-)
6. ClpP RNAi2 (+)
16SrRNA
12SrRNA
ND3
ND4/4L
ND5
ND6
COXI
COXIII
ATP6/8
CytB

Figure S2. Expression of *Drosophila* ClpP protease in Schneider cells. Schneider cells with no plasmid (control) or carrying pMt/invGFP/Hy (GFP RNAi) or pMt/invClpP 2/Hy (ClpP RNAi 2) were cultured for 5 d in the presence or absence of 0.1 mM CuSO4. (A) Protein extracts (10 μg) were fractionated by 4–12% SDS-PAGE, transferred to PVDF filters and probed with antibodies against ClpP, Lon, mtTFB2/TFB2M, ATPase α, or β tubulin as indicated. (B) Relative protein ratios were quantitated as described in Fig. 1B. The results represent the mean±SD of three independent experiments. *P<0.05; **P<0.01; ***P<0.001; versus control cells without induction (Student's t-test). (C) The relative ratio of mRNAs. Individual transcripts were analyzed by TaqMan-based real-time PCR assays. The ratio was determined as described in Fig. 1D. The results represent the mean±SD of three independent experiments and *P<0.05; **P<0.01 versus control cells without induction. (D) Increase of unprocessed COXIII-ATP6/8 and Cytb-ND6 transcripts in ClpP-depleted Schneider cells. Semi-quantitative RT-PCR analysis was performed as described in Fig. S1B. (E) TaqMan-based real-time PCR assays of mitochondrial gene transcripts. Data were normalized as described in Fig.S1A. The results represent the mean±SD of three independent experiments and *P<0.05; **P<0.01; versus control cells without induction.

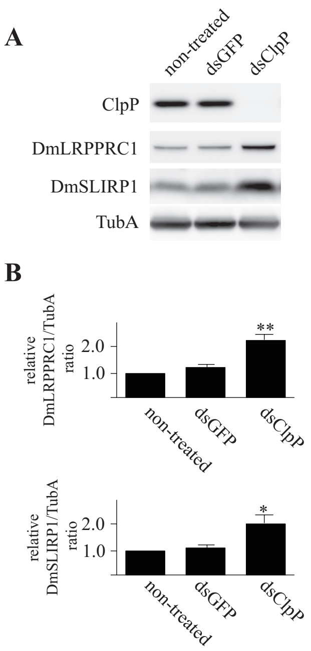

Figure S3. Transient depletion of ClpP in Schneider cells. Schneider cells were cultured for 6 d in the absence (non-treated) or presence of dsRNA of DmClpP (dsClpP) or GFP (dsGFP). (A) Immunoblot analysis was carried out as described in the legend to Fig. 1A. (B) The relative DmLRPPRC1 or DmSLIRP1 protein ratio with or without dsRNA treatment. Relative ratio of each cell lines was determined as described in Fig. 1B and normalized by the ratios of non-treated cells. The results represent the mean±SD of three independent experiments and *P<0.05; **P<0.01 versus untreated cells.

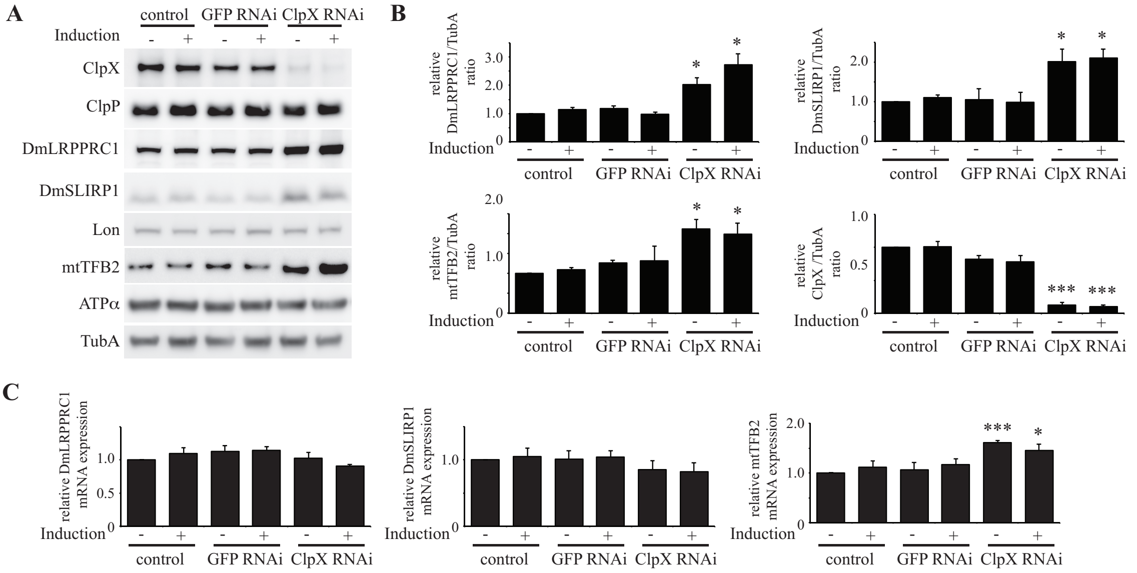

Figure S4. Protein levels of DmLRPPRC1 and DmSLIRP1 in ClpX-depleted Schneider cells. Schneider cells with no plasmid (control) or carrying pMt/invGFP/Hy (GFP RNAi) or pMt/invClpX/Hy (ClpX RNAi) were cultured for 5 d in the presence or absence of 0.1 mM CuSO4. (A) Immunoblot analysis was carried out as described in the legend to Fig. 1A. (B) The relative protein ratio was determined as described in Fig. 1B. The results represent the mean±SD of three independent experiments and *P<0.05; ***P<0.001 versus control cells without induction. (C) The relative mRNA ratio. The relative mRNA ratio was determined as described in Fig. 1D. The results represent the mean±SD of three independent experiments and *P<0.05; ***P<0.001 versus control cells without induction.

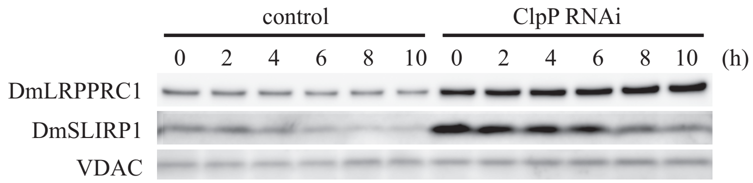

Figure S5. Cycloheximide chase assay of DmLRPPRC1 protein. Schneider S2 cells with no plasmid (control) or carrying pMt/invClpP/Hy (ClpP RNAi) were cultured in the presence of 200 μg/ml emetine and 100 μg/ml cycloheximide. The cells were isolated at indicated times. Total cell extracts were analyzed by immunoblot as described in the legend to Fig. 1A. The results are representative of two independent experiments.

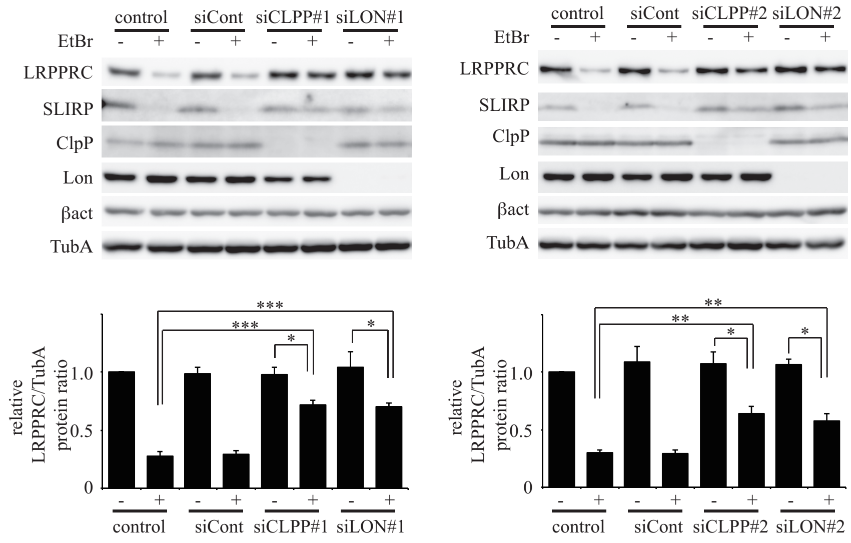

Figure S6. Dynamics of LRPPRC1 proteins during mitochondrial transcript depletion in ClpP or Lon knockdown HeLa cells. Three days after the first siRNA transfection, cells were re-transfected. Six hours after the second transfection, the medium was replaced with new medium with or without 50 ng/ml ethidium bromide and cultured for another 3 days. *Upper panel*, immunoblot analysis of HeLa cell extracts from non-transfected (control) or transfected with siRNA for non-target control (siCont), CLPP (siCLPP) or LONP1 (siLon). *Lower panel*, relative protein ratios of LRPPRC. Ratios were quantitated by normalizing LRPPRC protein levels to a tubulin protein levels. The results represent the mean±SD of four independent experiments (*P<0.05; **P<0.01; ***P<0.001, Student's t-test).

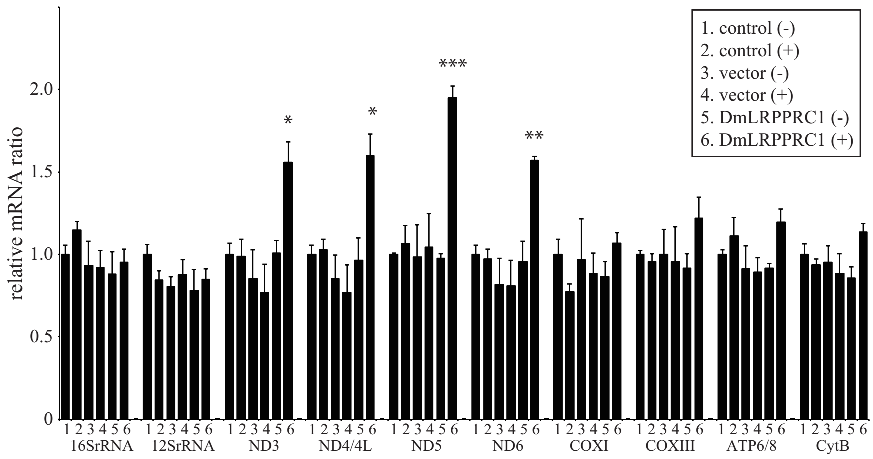

Figure S7. Effects of mitochondrial transcripts in DmLRPPRC1-overexpressing cells. Schneider cells with no plasmid (control) or carrying pMt/Hy (vector) or pMt/DmLRPPRC1/Hy (DmLRPPRC1) were cultured for 5 d in the presence or absence of 0.1 mM $CuSO_4$. TaqMan-based real-time PCR assays of mitochondrial gene transcripts. The levels of the transcripts were standardized by nuclear encoded gene, α tubulin. Data were normalized to the expression level in control cells without induction for each RNA species. The results represent the mean±SD of three independent experiments and *P<0.05; **P<0.01; ***P<0.001; versus control cells without induction.

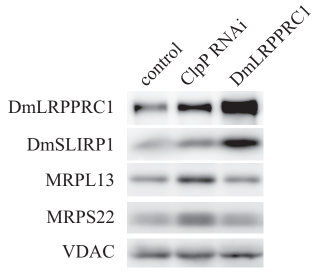

Figure S8. Immunoblot analysis of mitochondrial extracts. Immunoblot analysis of mitochondrial extracts from Schneider S2 cells (control), ClpP knockdown (ClpP RNAi) cells and DmLRPPRC1 overexpressing cells (DmLRPPRC1). Immunoblot analysis was carried out as described in the legend to Fig. 1A.

For Figure 1A

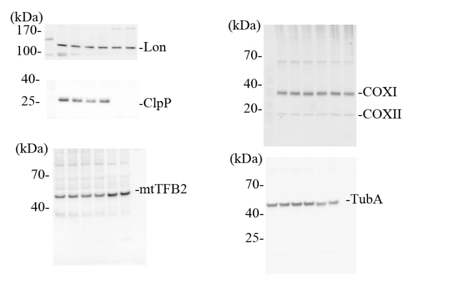

For Figure 1C

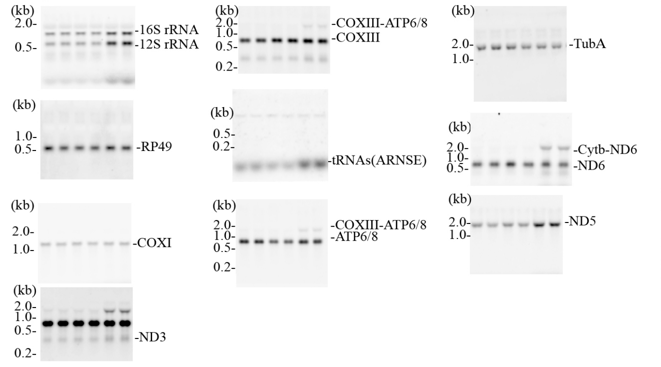

For Fig. 2A

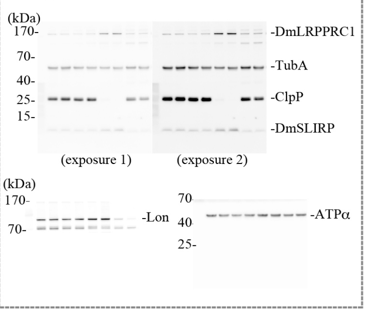

For Fig. 3A

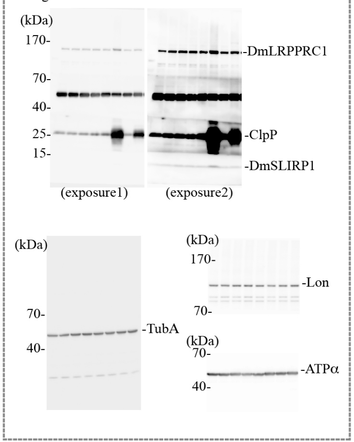

For Figure 4A

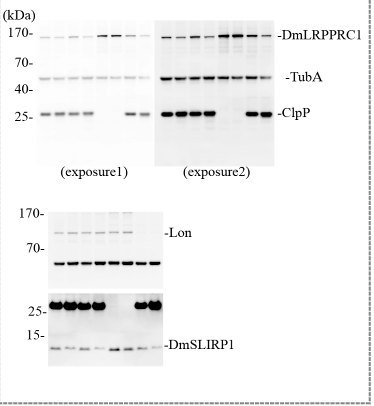

For Figure 4B

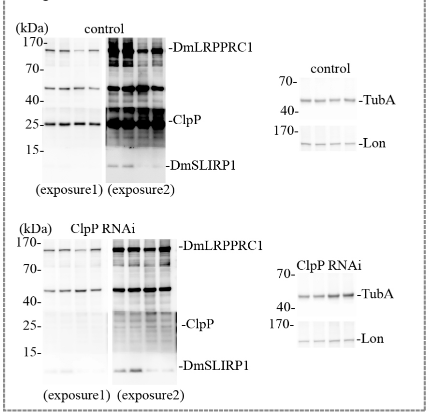

For Figure 5

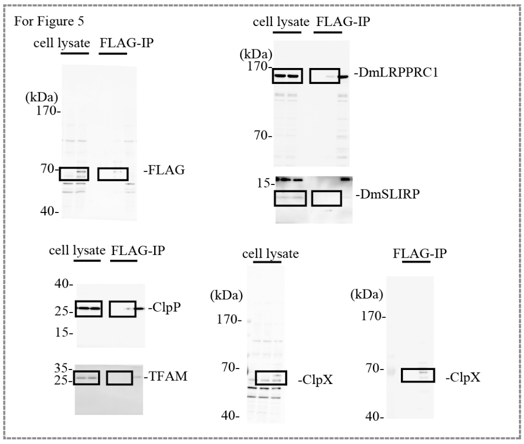

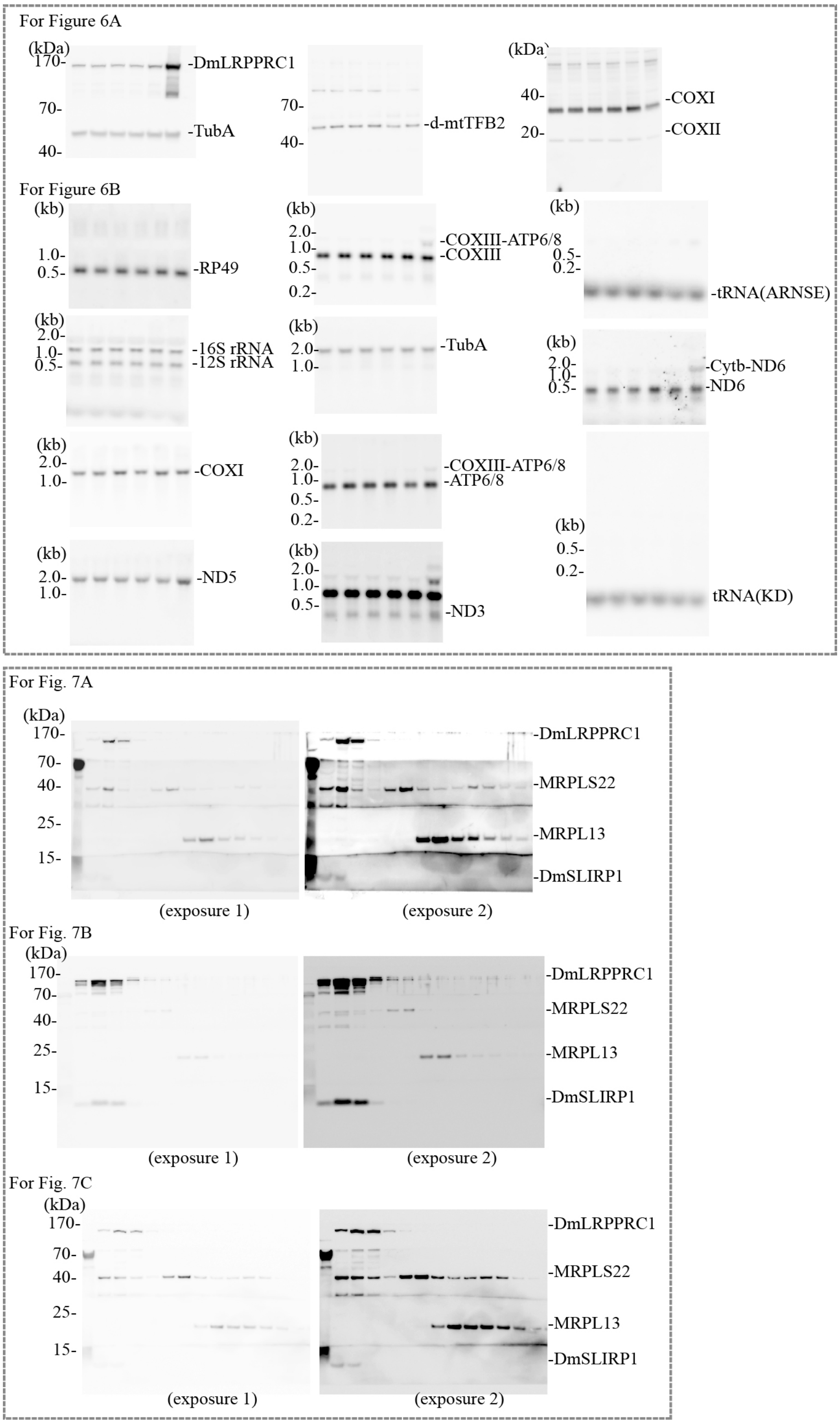

Figure S9. Blot images of Figures. Full-length blots used for Figure 1-7.

Supporting table 1. Primers for vector construction, dsRNA synthesis, RT-PCR analysis.

pMt/invClpP/Hy	5'	GCGCCTCGAGCAAAACCAGGATCATGCTGAA
	3'-1	GCGCGAATTCGGGATCGATCATTGCAACTTGGTGCGTTG
	3'-2	GCGCGAATTCAAAAAGCTTCATTGCAACTTGGTGCGTTG
pMt/invGFP/Hy	5'	GCGCACTAGTCTCGAGGGAGAAGAACTTTTCACTGG
	3'-1	GCGCGAATTCGGGATCGATTCTGCTAGTTGAACGCTTC
	3'-2	GCGCGAATTCAAAAAGCTTTCTGCTAGTTGAACGCTTC
pMt/invClpP 2/Hy	5'	GCGCCTCGAGACTAGTCAAAACCAGGATCATGCTGAA
	3'-1	GCGCGGATCCGGGATCGATCGGTGGCTTGACGTACTGCAT
	3'-2	GCGCGGATCCAAAAAGCTTCGGTGGCTTGACGTACTGCAT
pMt/invClpX/Hy	5'	GCGCACTAGTCTCGAGGAGCTGGTCAAACACATGGC
	3'-1	GCGCGAATTCGGGATCGATAGCTGACAAAGGTCTCAACC
	3'-2	GCGCGAATTCGGGATCGATAGCTGACAAAGGTCTCAACC
pMt/ClpP/Hy	5'	GCGCCTCGAGCAAAACCAGGATCATGCTGAA
	3'	GGGACTAGTTTACAAGTCTTCTTCAGAAATAAGCTTTTGTTCAATTGTTTCGAACTTATGATTG
S124A mutagenesis primers	Fw	GGATCCCATCGCGCAGGCCTGTC
	Rev	GACAGGCCTGCGCGATGGGATCC
pMt/ClpxX-FLAG/Hy	5'	GCGCCTCGAGCAGCAGCCACCATGAGCATG
	3'	GGGACTAGTTCATTTGTCGTCATCGTCCTTGTAGTCGATGTCGTGATCCTTATAGTCGCCATCGTGATCCTTGTAATCCTGTTTTAGTCGTACTTTCTCAC
dsRNA T7 ClpP	Fw	GTAATACGACTCACTATAGGGCATTGCAACTTGGTGCGTTG
	Rev	GTAATACGACTCACTATAGGGGAATTATTCAAATTGTTTCGAAC
dsRNA T7 DmLRPPRC1	Fw	GTAATACGACTCACTATAGGGCATGGCATCCATCCTGAGGACG
	Rev	GTAATACGACTCACTATAGGGGACATTTTCGTTGACGGGTAGAC

dsRNA T7 DmSLIRP1	Fw	GTAATACGACTCACTATAGGGACCTCATTTAGCAAAACCGAGTCC
	Rev	GTAATACGACTCACTATAGGGAAGTGGGGAATCTAAGATTTCTG
dsRNA T7 GFP	Fw	GTAATACGACTCACTATAGGGGGAGAAGAACTTTTCACTGG
	Rev	GTAATACGACTCACTATAGGGTCTGCTAGTTGAACGCTTC
RT-PCR ATP6/8	Fw	TCATTTAGTTCCTCAAGGAAC
	Rev	CGAACAGCTAATGTTCCAGG
RT-PCR COXIII	Fw	ATTAACAGGAGCTATCGGAGC
	Rev	TCTCGTGATACATCTCGTCATC
RT-PCR Cytb	Fw	AGCTATACATTACACAGCTG
	Rev	TTCCTCGTCCTACATGTAAG
RT-PCR COXIII-ATP6/8	Fw	GTATTAGAATCAGCTGTAGC
	Rev	ATGGTCATGGACTATAATCC
RT-PCR Cytb-ND6	Fw	GGACCTATTCGAATAATATC
	Rev	AATGATGCACCGTTAGCATG

Supporting table 2.

mtTFB2	Forward Primer	CGCCACCCATATACATACATCTC
	Probe	TGCAACAACGAGATTGGCTACCTCA
	Reverse Primer	GGCACCTTGGCTATGAATTTG
DmLRPPRC1	Forward Primer	AAAGTCTACCCGTCAACGAAA
	Probe	ACTCGCAGGCCAATGATTTGGAGT
	Reverse Primer	TGCATCATCACTCCCAAGATAC
DmSLIRP1	Forward Primer	GACATGTCGGACAGCGATAC
	Probe	AGCAGTCAAACAGCATTGGTTCCG
	Reverse Primer	GCCTGCTTGTAGTATCCGTAAA
TubA	Forward Primer	CCTCGAAATCGTAGCTCTACAC
	Probe	CCTTGTCGCGTGTGAAACACTTCC
	Reverse Primer	CAGCCTGACCAACATGGATA
mt-16SrRNA	Forward Primer	TTGGGTGTAGCCGTTCAAAT
	Probe	TCTTACATGATCTGAGTTCAAACCGGT
	Reverse Primer	AGATAGAAACCAACCTGGCTTAC
mt-12SrRNA	Forward Primer	GATAATCCACGATGGACCTTACT
	Probe	TTTGTAATCAGTTTATATACCGTCGTTATCAGAA
	Reverse Primer	ACTTAAATATAAGCTACACCTTGATCTG
mt-ND3	Forward Primer	AAAGCTTTAATCGACCGAGAA
	Probe	CCCATTTGAATGTGGATTTGATCCAAA
	Reverse Primer	GGTAGAATTAATGCAATCTCTACATC
mt-ND4	Forward Primer	ACTTATTGAGGTTTATGTGGTTCTTATAC
	Probe	TGCTCATGGTTTATGTTCTTCTGGGT

	Reverse Primer	CTTCGACTTCCAAGACGTTCA
mt-ND5	Forward Primer	CAAGCAATAGAAAGAAGTAAAGCTACA
	Probe	CCCAATTCGATTAGATAACGCAGTTAATATACCAGC
	Reverse Primer	GGGTGAGATGGTTTAGGACTTG
mt-ND6	Forward Primer	ATATTATAATTTGTACCATTCAA
	Probe	AATCATTACCATGAGTACGAATTATAGAAACT
	Reverse Primer	GACATTTAGAGTATGTGAAG
mt-COXI	Forward Primer	CCGTTGGAATAGATGTAGATACTCG
	Probe	ACATGGAACTCAACTTTCTTATTCTCCAGCT
	Reverse Primer	CAACTCCTGTTAATCCTCCTACTG
mt-COX3	Forward Primer	CCATGACCATTAACAGGAGCTA
	Probe	TCGGAGCTATAACAACTGTATCAGGT
	Reverse Primer	CTCGTGATACATCTCGTCATCAT
mt-CytB	Forward Primer	GTCGAGACGTTAATTATGGTTGATTAT
	Probe	ACGAACTTTACATGCTAACGGTGCA
	Reverse Primer	CCGTAATAAATTCCTCGTCCTACA
mt-ATP6/8	Forward Primer	CTCAAGGAACACCCGCTATT
	Probe	TCGACCTGGAACATTAGCTGTTCGA
	Reverse Primer	AATGTCCAGCAATTATATTAGCAGTTA
